# Calibration curves by ^60^Co with low dose rate are different in terms of dose estimation – a comparative study

**DOI:** 10.1590/1678-4685-GMB-2018-0370

**Published:** 2020-02-17

**Authors:** Mariana Esposito Mendes, Julyanne Conceição Goes de Mendonça, Suy Hwang, Marina Di Giorgio, Fabiana Farias de Lima, Neide Santos

**Affiliations:** 1 Universidade Federal de Pernambuco, Departamento de Genética, Recife, Pernambuco, Brazil.; 2 Centro Regional de Ciências Nucleares do Nordeste, Recife, Pernambuco, Brazil.; 3 Autoridad Regulatoria Nuclear, Ciudad de Buenos Aires, Argentina

**Keywords:** Dicentrics, gamma radiation, biological dosimetry, intercomparisons

## Abstract

Biological dosimetry aims to estimate individual absorbed doses due ionizing radiation exposure. The dicentric chromosomes are considered the most specific biomarker for dose estimation. This study aimed to compare calibration curves for linear low energy transfer (LET) radiation built from low dose rates and whether they vary in terms of dose estimation. For that we did a search in the literature of all calibration curves produced with low dose rates and we simulated the dose estimation from pre-established dicentric’s frequencies. The information on methodologies and cytogenetic results of each study were analyzed. As expected dose rate influence β coefficients, especially at higher doses. However, we have seen that some doses were not statistically different but they should be, because there is a significant association between the productions of dicentrics and dose rate. This comparative study reinforced the robustness of the dicentric assay and its importance in biological dosimetry. We also emphasized that the dose rate was an important factor in dose estimations. Thus, intercomparison exercises should take into account the dose rates of the participating laboratories, because the dose rates might explain why some results of estimated doses fall outside the recommendations.

## Introduction

Biological dosimetry aims to estimate individual absorbed doses due ionizing radiation exposure. The determination of absorbed dose using a biological method is very important, because offers essential information that will support the medical management of patients in emergencies (Di Giorgio *et al.*, 2011; Gruel *et al.*, 2013).

Adequate dose estimation is based on biomarkers analysis that should be specific and sensitive to radiation, and independent of other environmental exposures. The international biodosimetry considers the dicentric chromosome assay as “gold standard” for recent radiation exposures, because in general, the yield of dicentrics is very low (~0.5–1.0 per 1000 cells) in natural occurrence. Besides that, dicentrics can be used to individual dose assessment for homogeneous whole-body exposures to doses as low as 100 mGy for low linear energy transfer (LET) radiation, if up to 1000 cells are analyzed. It is also possible differentiates between partial and whole body exposures, as well as high or low LET radiation (IAEA, 2011; Lee *et al.*, 2012; Pernot *et al.*, 2012; Wong *et al.*, 2013, Hall *et al.*, 2017).

Several studies have used dicentrics for dose estimation after accidental exposure of workers, such as two radiographers and their driver who were seriously exposed to an ^192^Ir industrial radiography source, that became detached from its wind-out cable (Sevan’kaev *et al.*, 2002), and technician involved in the maintaining of X-ray equipment (Thierens *et al.*, 2005). There are also major accidents, such as soldiers carrying small sources of ^137^Cs in their pockets, leading to partial and prolonged body exposures; employees involved in the ^235^U enrichment process were exposed to gamma rays and neutrons; and radiotherapy breast cancer patients were undergoing were exposed to electrons, which reached about 100 Gy (Wojcik *et al.*, 2004). As well as large-scale radiation accidents, such as Chernobyl, Ukraine, in 1986 (Hatch *et al.*, 2005; Beresford and Copplestone, 2011), Goiânia, Brazil, in 1987 (Ramalho *et al.*, 1988, 1991), Fukushima, Japan, in 2011 (Beresford and Copplestone, 2011; Yasunari *et al.*, 2011; Gering *et al.*, 2013).

The establishment of at least one appropriate calibration curve is an essential condition for dose estimation. It is necessary to build a pre-defined dose-response calibration curve, where the yields of chromosomal aberrations are dose related by the linear quadratic equation for low LET, and by the linear equation for high LET radiation. The standard curve to be used must have a radiation quality equal to or very similar to the specific type of radiation involved in the emergency (IAEA, 2011; Roy *et al.*, 2012). The International Atomic Energy Agency (IAEA, 2011) recommends that each biodosimetry laboratory define its own calibration curve, as intrinsic differences in protocols and dose interpretation using a calibration curve produced elsewhere may introduce extra uncertainty in dose estimation.

Most *in vitro* calibration curves built and published refer to acute exposure using dose rates of 0.5 Gy/min. However, major accidental exposure scenarios involve irradiation for an uncertain period varying distances and dose rates (Vinnikov *et al.*, 2010; IAEA, 2011). However, there is one mathematical model for estimating dose for accidents involving a lower dose rate employing the time-dependent factor known as G-function (Lea and Catcheside, 1942 *apud* IAEA, 2011). This time-dependent factor is use to modify the dose squared coefficient and thus allow for the effects of dose protraction (IAEA, 2011). However, some biodosimetry groups have chosen to produce a non-acute calibration curve in order to better understanding how the quadratic coefficient should be modified to interpret aberration yields in accidents involving prolonged irradiation.

In this context, this study aimed to compare calibration curves for linear low energy transfer (LET) radiation built from low dose rates and whether they vary in terms of dose estimation. For that, we performed a search in the literature of all gamma radiation calibration curves produced at low dose rates and simulated dose estimation from pre-established dicentrics frequencies.

## Materials and Methods

### Construction of calibration curve

Firstly, we built a reference calibration curve for our laboratory. For this purpose, blood samples (5 mL) were collected from a voluntary non-smoking woman with informed consent (ethics approval no. 269.483). Each sample was irradiated with Cobalt60 irradiator (Gammacell 220 ® - MDS Nordion, Ottawa, Canada) with average energies of 1.25 MeV at Departamento de Energia Nuclear (DEN-UFPE, Recife, Brazil). With dose rates of 0.055 to 0.048 Gy/min with uncertainty of 2% at the point of irradiation. The blood sample tubes were wrapped in 4 mm of dense material (following IAEA, 2011). For the elaboration of the calibration curve, blood samples were irradiated between 0.15 - 5 Gy, and after incubated for 2 h at 37 ºC.

Heparinized whole blood (0.5 ml) were culture for 48h in 4 mL of RPMI-1640 (Sigma) medium supplemented with 0.2 mL of phytohaemagglutinin (Sigma), and 1 mL of fetal bovine serum (Biological Industries). In addition, 0.1 mL of 0.0016% colchicine (Sigma) was added 46 hours after culture started. At the end of 48 h, the supernatant was removed, and the cell pellet homogenized in 8 mL of 0.075M KCl, and placed at 37 ^o^C for 20 min, after the supernatant was removed and cells fixed in 7 mL Carnoy’s fixative solution (3:1 methanol: glacial acetic acid mixture). Finally, chromosomal preparations were stained with a 5% Giemsa stain in pH 6.8 buffer for 6 min. We also followed the IAEA (2011) recommendation that only complete metaphases be recorded, i.e. those with 46 centromeres and if the cell contains unstable aberrations, then it should balance. Therefore, if a spread containing a dicentric should also have an acentric fragment, yet still count to 46 pieces.

### Selected calibration curves

For this comparative study, we made an exhaustive literature search of experimental studies on Web of Science, relying on the following keywords: “dosimetry”, “dose response”, “calibration curve”, “gamma radiation” and “dicentrics”. Once we located these papers, we tested if they fulfilled our inclusion criteria. We also searched the reference lists of all identified publications in an attempt to locate additional publications. We included (1) calibration curves already defined by selected cobalt-60 sources at dose rates below 0.5 Gy/min and a single curve was used as the standard (dose rate 0.5 Gy/min); (2) studies that presented all information about dicentric frequencies; (3) dose effect curves established by manual scoring. Exclusion criteria were (1) studies that presented only information on dicentric plus ring frequencies; (2) studies without dicentric distribution per cell; (3) studies in which dose effect curves were established by automatic or semiautomatic scoring. Until April 10, 2019, we found seven studies following these criteria. Then, we compared information on methodologies (dose analysis, scored cells) and cytogenetic results from each study (frequencies and distribution of dicentrics).

### Comparison of Calibration Curves

All selected calibration curves were compared using two approaches: 1) the parameters of each methodology, as well as dicentrics distribution data with no modifications; and 2) all data (including statistic coefficients) were standardized using Dose Estimate software (Ainsbury *et al.*, 2010) to perform the statistics. Thus, all results were re-tested to analyze their conformity with Poisson distribution by means of the Papworth’s u test (Acharya *et al.*, 2009; IAEA, 2011). Thereafter, using R-based tools2 (R Development Core Team, 2012) the Pearson’s Chi-squared test was applied to determine whether the yield of dicentric varies according to calibration curves.

Firstly, we simulate the dose estimation from six pre-determined dicentric frequencies between 0.02-1 dicentric per cell. Thus, all results of estimated absorbed doses along with their lower and upper confidence limits were used. The confidence limits were based on exact Poisson error on yield, because this simplified method is more commonly encountered in biodosimetry comparison exercise (Szluinska *et al.*, 2007).

In addition, we used ANOVA test to compare dose estimation among all calibration curve. ANOVA is a parametric method of analysis, which is generally applied to normal data, but the data type distributed by Poisson also approximates normal distribution sufficiently to ensure that ANOVA can be applied. When performing a variance analysis, the null hypothesis considered is that there is no difference in treatment averages; consequently, in these cases, there is no difference between the estimated doses (IAEA, 2011; Wilkins *et al.*, 2015). Subsequently, Tukey’s multiple comparison tests were conducted to find significantly different media.

## Results

The distributions of dicentric cells selected for our calibration curve followed a Poisson distribution (Table 1), and the curve was adjusted to linear-quadratic model (Table 2) as expected for blood samples homogeneously exposed to low LET gamma-radiation (IAEA, 2011). A tendency toward under-dispersion was observed in the distributions of dicentric cells, but this trend was not statistically significant, except at the 3 Gy dose (Table 1).

**Table 1:** Distribution of dicentric chromosomes produced using low dose rate with respective dispersion indexes and u values from this work.

Distribution of dicentrics											
Dose (Gy)	Cells scored	Dicentrics	0D	1D	2D	3D	4D	5D	Yield	σ^2^/y	U
0	4571	5	4566	5					0.001	0.999	-0.047
0.15	2029	10	2019	10					0.005	0.996	-0.149
0.25	1004	7	997	7					0.007	0.994	-0.145
0.5	1006	18	988	18					0.018	0.983	-0.39
0.75	1000	33	967	33					0.033	0.968	-0.727
1	1000	59	941	59					0.059	0.942	-1.31
1.5	1000	80	923	74	3				0.08	0.996	-0.09
2	490	93	406	75	9				0.190	1.010	0.091
3	223	100	135	76	12				0.448	0.795	-2.17
4	136	103	61	55	14	4	2		0.757	0.988	-0.101
5	80	112	19	30	19	6	4	2	1.4	1.06	0.377

(σ^2^) variance; (y) mean; (U) u-test.

**Table 2:** Comparison of methodologies and coefficient values of calibration curve produced by cobalt-60 sources with low dose rates.

References	Country	Volunteers Number	Colcemid time (h)	BrdU	Dose range (Gy)	Dose rate (Gy/min)	Dose rate/0.5 (Gy/min)	C	SE	α	± SE (Gy^-1^)	β	± SE (Gy^-2^)
Bauchinger *et al.* (1983)	Germany	1 (M)	**-**	Yes	0.5 – 4	0.017	3%	0.0009	0.0009	0.0095	0.0064	0.0415	0.0035
Schmid *et al.* (2002)	Germany	1 (W)	44	Yes	0.25 – 4	0.033	7%	0.0003	0.0002	0.0139	0.0052	0.0304	0.0030
This work	Brazil	1 (W)	46	No	0 – 5	0.055-0.048	11%	0.0014	0.0008	0.0074	0.0069	0.0449	0.0044
Martins *et al.* (2013)	Portugal	16	47	No	0 – 3	0.18-0.13	36%	0.0011	0.0006	0.0098	0.0036	0.0489	0.0020
Lindholm *et al.* (1998)	England	2 (1M and 1W)	45.5	No	0 – 5	0.24	48%	0.0006	0.0003	0.0136	0.0045	0.0542	0.0035
Top *et al.* (2000)	Turkey	3 (2M and 1W)	45	Yes	0 – 5	0.425	85%	0.0007	0.0008	0.0070	0.0067	0.0601	0.0034
Köksal *et al.* (1995)	Turkey	1 (W)	45	Yes	0.98 – 4.89	0.4573	91%	0.0005	0.0003	0.0216	0.0060	0.0706	0.0025
Lloyd *et al.* (1986)	England	a panel	45	Yes	0.05 – 5.05	0.5	100%	0.0004	0.0009	0.0145	0.0060	0.0760	0.0030

(-) Not informed; (SE) Standard error. Model equation y = C+αD+β^2^D.

All compared studies were performed with different parameters, based on the different methodologies used in building each calibration curve. Consequently, these curves had different coefficient values (Table 2). Most of these studies used 48 h for the lymphocyte cultures, and a few studies did not use bromodeoxyuridine (BrdU). Other differences between studies included the irradiation conditions (low dose rates and dose ranges) and the number of volunteers. We did not consider differences in slide scoring criteria, because that information was not available in most selected studies.

To reduce the differences between the coefficients values used for curve fitting in statistical analyses, we decided to re-analyze all published calibration curves with a single software program (Dose Estimate). After this re-analysis, we found minimal differences between the newly generated coefficients and the coefficients previously published, with the exception of one linear coefficient (α) from the study by Top *et al.* (2000) (Table 2).

The frequencies of dicentrics increased with the absorbed dose and with the dose rate; these same tendencies were observed in the distributions of dicentrics per cell. To confirm this behavior, we performed the Pearson’s Chi-squared test. We compared only doses of 0.5, 1, and 2 Gy, because these doses were included in all calibration curves. We found a significant association between production of one dicentric per cell at doses of 1 and 2 Gy and in cases of more than one dicentric per cell, we found significant differences among dose rates, mainly at the 2 Gy dose. This finding was confirmed with a Chi-squared test (Table S1).

To evaluate whether these differences in dose rates generated diverse estimated doses, we analyzed six different dicentric frequencies (0.02 – 1 dicentric per cell) that resulted in absorbed doses of 0.5 to 5 Gy (Table 3). As expected, we observed two major trends: (1) as the dicentric frequencies increased, the estimated doses and their uncertainties (SE) increased, particularly for curves that included included the lower dose rate; and (2) in studies with the highest dose rates, the number of dicentrics could predict the lowest absorbed doses (Table 3).

**Table 3:** Comparison of estimated absorbed doses by selected calibration curves using Dose Estimate software.

References	Bauchinger *et al.* (1983)	Schmid *et al.* (2002)	This work	Martins *et al.* (2013)	Lindholm *et al.* (1998)	Top *et al.* (2000)	Köksal *et al.* (1995)	Lloyd *et al.* (1986)
Country	Germany	Germany	Brazil	Portugal	England	Turkey	Turkey	England
Culture time (h)	48	47	48	48	48	48	48	48
Dose rate (Gy/min) / 0.5 Gy/min	3%	7%	11%	36%	48%	85%	91%	100%
Dicentric frequencies		Estimated doses ± 95% CL (Gy)						
0.02a	0.574	0.608	0.566	0.530	0.486	0.511	0.394	0.421
	(0.420	(0.438	(0.415	(0.387	(0.354	(0.383	(0.282	(0.310
	0.743)	0.800)	0.732)	0.687)	0.633)	0.653)	0.521)	0.545)
0.15b	1.784	2.002	1.739	1.648	1.540	1.519	1.310	1.311
	(1.632	(1.825	(1.592	(1.507	(1.407	(1.392	(1.194	(1.199
	1.943)	2.186)	1.891)	1.794)	1.678)	1.651)	1.431)	1.428)
0.2c	2.079	2.345	2.022	1.919	1.797	1.764	1.535	1.528
	(1.927	(2.167	(1.876	(1.779	(1.664	(1.637	(1.419	(1.416
	2.236)	2.528)	2.174)	2.064)	1.934)	1.895)	1.655)	1.644)
0.7d	3.991	4.574	3.863	3.982	3.469	3.353	2.998	2.940
	(3.511	(4.013	(3.400	(3.238	(3.048	(2.954	(2.630	(2.585
	4.501)	5.169)	4.353)	4.151)	3.915)	3.777)	3.389)	3.316)
0.75e	4.136	4.743	4.002	3.815	3.595	3.473	3.109	3.047
	(3.655	(4.181	(3.539	(3.371	(3.174	(3.073	(2.740	(2.691
	4.644)	5.336)	4.491)	4.283)	4.040)	3.896)	3.498)	3.422)
1f	4.793	5.510	4.634	4.421	4.170	4.020	3.613	3.533
	(4.312	(4.949	(4.172	(3.977	(3.750	(3.620	(3.244	(3.177
	5.298)	6.100)	5.120)	4.886)	4.612)	4.439)	4.000)	3.906)

(95% CL) 95% Confidence limits from exact Poisson error on yield; (a) 20 dic/1000cells; (b)150 dic/1000 cells; (c) 200 dic/1000 cells; (d) 70 dic/100 cells; (e) 75 dic/100 cells; (f) 100 dic/100 cells;

First, the ANOVA results showed that the estimated doses were different at almost all frequencies tested (the first column number of groups compared in Table 4). Second, Tukey´s test, applied only when the ANOVA results showed a significant difference, indicated significant differences among calibration curves (Table 5), principally the absorbed doses from Schmid *et al.* (2002) with Köksal *et al.* (1995) and Lloyd *et al.* (1986).

**Table 4:** Comparison results of estimated absorbed doses by ANOVA after jackknife-like resampling method.

		Identification number of groups compared											
		1	2	3	4	5	6	7	8	9	10	11	12
Dose rate (Gy/min) /0.5 Gy/min	3%	Bauchinger *et al.* (1983)	Bauchinger *et al.* (1983)						Bauchinger *et al.* (1983)	Bauchinger *et al.* (1983)	Bauchinger *et al.* (1983)	Bauchinger *et al.* (1983)	Bauchinger *et al.* (1983)
	7%	Schmid *et al.* (2002)											
	11%	This work	This work	This work					This work	This work	This work	This work	This work
	36%	Martins *et al.* (2013)	Martins *et al.* (2013)	Martins *et al.* (2013)	Martins *et al.* (2013)				Martins *et al.* (2013)	Martins *et al.* (2013)	Martins *et al.* (2013)	Martins *et al.* (2013)	
	48%	Lindholm *et al.* (1998)	Lindholm *et al.* (1998)	Lindholm *et al.* (1998)	Lindholm *et al.* (1998)	Lindholm *et al.* (1998)			Lindholm *et al.* (1998)	Lindholm *et al.* (1998)	Lindholm *et al.* (1998)		
	85%	Top *et al.* (2000)	Top *et al.* (2000)	Top *et al.* (2000)	Top *et al.* (2000)	Top *et al.* (2000)	Top *et al.* (2000)		Top *et al.* (2000)	Top *et al.* (2000)			
	91%	Köksal *et al.* (1995)	Köksal *et al.* (1995)	Köksal *et al.* (1995)	Köksal *et al.* (1995)	Köksal *et al.* (1995)	Köksal *et al.* (1995)	Köksal *et al.* (1995)	Köksal *et al.* (1995)				
	100%	Lloyd *et al.* (1986)	Lloyd *et al.* (1986)	Lloyd *et al.* (1986)	Lloyd *et al.* (1986)	Lloyd *et al.* (1986)	Lloyd *et al.* (1986)	Lloyd *et al.* (1986)					
Frequencies of dicentrics	p-values by ANOVA												
	0.02	0.606	0.643	0.642	0.683	0.665	0.522	0.799	0.688	0.941	0.888	0.935	0.954
	0.15	<0.001*	0.003*	0.009*	0.033*	0.090	0.127	0.992	0.014*	0.167	0.256	0.556	0.732
	0.2	<0.001*	<0.001*	0.003*	0.014*	0.050	0.089	0.945	0.005*	0.086	0.167	0.458	0.671
	0.7	0.008*	0.058	0.096	0.157	0.343	0.418	0.856	0.14	0.438	0.597	0.869	0.761
	0.75	0.005*	0.051	0.096	0.187	0.318	0.399	0.848	0.127	0.408	0.544	0.72	0.752
	1	0.001*	0.019*	0.05	0.106	0.220	0.320	0.804	0.062	0.277	0.433	0.648	0.708

(*) *p*-value < 0.05 means that the estimated doses are not statistically similar.

**Table 5:** Results of Tukey’s test after comparing all possible pairs of means.

Tukey multiple comparisons of means		Bauchinger *et al.* (1983) versus Köksal *et al.* (1995)	Bauchinger *et al.* (1983) versus Lloyd *et al.* (1986)	Schmid *et al.* (2002) versus Martins *et al*. (2013)	Schmid *et al.* (2002) versus Lindholm *et al.* (1998)	Schmid *et al*. (2002) versus Top *et al.* (2000)	Schmid *et al.* (2002) versus Köksal *et al.* (1995)	Schmid *et al.* (2002) versus Lloyd *et al.* (1986)	This work versus Köksal *et al.* (1995)	This work versus Lloyd *et al.* (1986)	Martins *et al.* (2013) versus Köksal *et al.* (1995)	Martins *et al.* (2013) versus Lloyd *et al.* (1986)
Dose rate (Gy/min)/0.5 Gy/min		3% - 91%	3% - 100%	7% - 36%	7% - 48%	7% - 85%	7% - 91%	7% - 100%	11% - 91%	11% - 100%	36% - 91%	36% - 100%
Frequencies of dicentrics	Identification number of groups compared	p-values by Tukey’s test										
0.15	1	0.015	0.015		0.019	0.013	0.0004	0.0004	0.032	0.033		
	2	0.011	0.011						0.022	0.022		
	3								0.018	0.018		
	4										0.056	0.057
	8	0.013							0.025			
0.20	1	0.005	0.004	0.033	0.004	0.002	0.00007	0.00006	0.012	0.011		0.058
	2	0.003	0.003						0.008	0.007	0.045	0.040
	3								0.007	0.006	0.035	0.031
	4										0.028	0.025
	8	0.004							0.010		0.047	
0.70	1						0.012	0.008				
0.75	1					0.053	0.008	0.006				
1	1		0.054		0.036	0.016	0.002	0.001				
	2	0.056	0.037									

Note. Here were only show the comparisons of curves in Tukey’s test that has a p-value <0.05. Therefore it is the doses that differ statistically and had p-value <0.05 in the ANOVA test shown in Table 4.

To gain a better understanding of the sources of bias, including the potential effects of outlier curves in dose estimations, we replicated our analysis with a jackknife-like resampling method. First, absorbed doses from Schmid *et al.* (2002) were withdrawn from the analysis group. However, the significant differences between estimated doses persisted (Tables 4 and 5) until some estimated doses did not present statistical significance (Table 4).

All compared estimated doses and their respective uncertainties were not significantly different at the 0.02 dicentric frequency. On the other hand, at the intermediate frequencies of 0.15 and 0.20 dicentrics per cell, we observed more conflicts (*p*-values < 0.05) than we observed at the more elevated frequencies. We speculated that this result was influenced by the 95% confidence limits, because the uncertainties were low in dose estimates at intermediate frequencies, which were defined with exact Poisson errors (Tables 4 and 5).

## Discussion

Dicentric chromosomes represent specific, sensitive biomarkers for estimated absorbed doses. Indeed, this chromosomal aberration appears, regardless of the protocol used for dose estimation (Table 2) (Lee *et al.*, 2012; Wong *et al.*, 2013). When dose-response curves are constructed, interlaboratory differences might well occur, due to inherent protocol differences. The differences in coefficients might be explained by several factors, including the total absorbed dose, dose rate, irradiated cell lines, biological endpoints, LET, and energy (IAEA, 2011; Lee, 2011; Okumura *et al.*, 2013). Another factor that might affect the results is the number of volunteers used in constructing the calibration curves, because inter-individual variability might affect the results. However, until recently, the construction of curves based on individual variability of dicentric chromosomes have not been well studied. Martins *et al.* (2013) produced calibration curves based on data from sixteen donors. The donors were distributed by age and gender to examine potential effects on any differences observed. However, in general, all the curves constructed showed good fits to the data. Although the fits were somewhat less accurate for data from females and from the oldest age group, but no significant differences were found.

Some variables are highly critical in constructing dose-response curves; e.g., the dose rate and the scoring criteria. The dose rate is a critical factor when analyzing chromosomal aberrations, because prolonged irradiation times reduce the frequency of dicentrics induced by low LET radiation. Extended irradiation times allow cellular repair mechanisms to correct the damage, which then reduces the frequency of dicentrics. To generate a dicentric, two lesions, one in the DNA double helix of each unreplicated chromosome, must be produced within a target zone. With low LET radiation, the probability is low that two ionizing events will occur in a single track. Therefore, two ionizations are necessary to cause damage in two chromosomes to produce a dicentric. Dicentrics produced by one irradiation will occur at a frequency proportional to a linear dose function (linear coefficient - α), and dicentrics induced by two irradiations will have a frequency proportional to the square of the dose (quadratic coefficient - β). When lesions are produced by two independent irradiations, and the dose rate is low, the likelihood is high that an injury produced by the first irradiation will be repaired before the second irradiation causes damage. Thus, the two lesions are unlikely to form a dicentric chromosome (Vinnikov *et al.*, 2010; IAEA, 2011).

Studies have shown that the linear coefficient (α) is independent of the dose rate, and that the quadratic coefficient (β) decreases as the dose rate decreases (Brewen and Luippold, 1971; Lloyd *et al.*, 1975, 1981; Bauchinger *et al.*, 1979, 1983). This behavior was also observed in the calibration curves compared in this study. We noted a reduction in the β term of the calibration curve as the dose rate decreased (Table 2).

However, the β coefficient of Schmid *et al.* (2002) behaved differently from the expected behavior. Those authors also noted that reductions in the β coefficient could not be attributed to a dose-rate effect; instead, other protocol aspects might have influenced this coefficient value, such as the scoring criteria. Scoring depends on two main factors (i) the spreading quality and metaphase selection, and (ii) the dicentric identification (Roy *et al.*, 2004). These factors might influence the criteria for identifying dicentrics, which could then increase or decrease the number of dicentrics considered in the total analysis.

In this comparison study, we observed the greatest divergences in intermediate dicentric frequencies, because the uncertainties in dose estimations were relatively small. At the higher dicentric frequencies, the respective uncertainties were larger; thus, the uncertainty in dosing contributed to the lack of significant differences (Szluinska *et al.*, 2007). We decided to perform an identical analysis with the ANOVA and Tukey’s test, except that we used the 95% confidence limits from combined Poisson and calibration curve errors. However, we observed the same performances as those mentioned in the Results section. Nevertheless, the differences among calibration curves were less pronounced, because the uncertainties in dose estimations were numerically higher. Consequently, in the comparison analysis, there were smaller differences among estimated doses (results shown Tables S2- S4).

In some cases, the estimated doses were not statistically different, but they should have been, because there is a significant association between the productions of dicentrics and dose rate (IAEA, 2011). Moreover, problems related to dose rate could also appear in intercomparison exercises. The propose of intercomparison exercises is to establish an operational network in biodosimetry for managing large numbers of potentially overexposed individuals, with mutual assistance, in cases of emergency (Oestreicher *et al.*, 2017). Intercomparison exercises can be performed with distinct methods and statistical analyses. For example, biodosimetry labs might receive irradiated blood samples to set up lymphocyte cultures, according to their own standard protocols (Beinke *et al.*, 2013; Wilkins *et al.*, 2015; Oestreicher *et al.*, 2017), or they might receive slides (Ramalho *et al.*, 1991; Roy *et al.*, 2004; Di Giorgio *et al.*, 2011; Liu *et al.*, 2016). The reported estimated doses per laboratory can be compared with the mean absolute difference (MAD), the z test, or an ANOVA (Di Giorgio *et al.*, 2011; Beinke *et al.*, 2013; Wilkins *et al.*, 2015). For intercomparison exercises, labs that only receive slides have a greater advantage, because slide samples avoid the effects of blood preparation; consequently, this sort of intercomparison is only affected by dicentric scoring. The results from intercomparison studies have indicated that this sort of exercise produced less variation among laboratories (Roy *et al.*, 2004; Di Giorgio *et al.*, 2011; Liu *et al.*, 2016).

Even some labs reporting higher frequencies of aberrations when the data are converted into absorbed doses with reference to the laboratory’s own calibration curve the inter-laboratory difference is removed (Grégoire *et al.*, 2013). The RENEB intercomparisons (Oestreicher *et al.*, 2017) caught our attention, because they included cases, where laboratories defined the frequency of dicentrics near to general dicentrics mean from other laboratories, but the estimated doses fell outside the recommended statistical criteria. The RENEB study was a global exercise that used dicentrics for dose assessments. A total of 42 laboratories from 31 countries all over the world participated. Blood samples were irradiated with ^137^Cs gamma rays (dose rate 0.495 Gy/min), and each laboratory was asked to set up at least two lymphocyte cultures per sample, according to their own standard protocols, with consideration of the IAEA recommendations (IAEA, 2011) and the International Organization for Standardization (ISO) standards (ISO 19238, 2014; ISO 21243, 2008). As expected, all calibration curves had a specific shape, but some curves had low β coefficients (those from L10, N8, N9, and N14 labs). Those laboratories showed very good agreement between the number of dicentrics detected in slides of 50 cells/slide; the numbers fell within the theoretically expected range, even though they used different lymphocyte cultures and scoring criteria. However, their estimated doses were above the recommended range, for both simulated doses (0.85 Gy and 2.7 Gy). Consequently, these intercomparison results corroborated our results, which showed that curves with lower β coefficients generated higher than expected estimated doses. Thus, the results generated were outside the recommended means, independent of the type of test used (i.e., the z test in this intercomparison study and the ANOVA in our comparison study).

Another interesting point of this intercomparison exercise was that the percentage of correct dose estimations did not change (81–76%) for the low dose-point (0.85 Gy) comparing only eighteen laboratories involved in the RENEB with 42 labs. However, in the intercomparison exercise, the percentage of correct dose estimations for the high dose-point (2.7 Gy) increased from 39 to 61%, when all labs were included. This increase in correct dose estimations might be due to the higher number of curves compared or to an increase in uncertainties, rather than improvements in lab performances.

## Conclusions

This comparative study reinforced the robustness of the dicentric assay and its importance in biological dosimetry. We also emphasized that the dose rate was an important factor in dose estimations. Thus, intercomparison exercises should take into account the dose rates of the participating laboratories, because the dose rates might explain why some results of estimated doses fall outside the recommendations.

## Supplementary material

The following online material is available for this article:

Table S1
*P*-values from Pearson’s Chi-squared test.

Table S2 Comparison of estimated absorbed doses by selected calibration curves using Dose Estimate software.

Table S3 Comparison results of estimated absorbed doses by ANOVA after jackknife-like resampling method.

Table S4 Results of Tukey’s test after compared all possible pairs of means.
