# Supplementary material for: Calibration curves by 60Co with low dose rate are different in terms of dose estimation – a comparative study
Source: Genet Mol Biol. 2020 Feb 17;43(1):e20180370. doi: 10.1590/1678-4685-GMB-2018-0370 (PMC7231543; doi:10.1590/1678-4685-GMB-2018-0370)
Supplement: Supplementary file 1 [file 1415-4757-GMB-43-1-e20180370-20200108-suppl1.pdf]

Genetics and Molecular Biology 42, 2, (2020)

Supplementary Material to “Calibration curves by 60Co with low dose rate are different in terms of dose estimation – a comparative study”

Table S1. P-values from Pearson's Chi-squared test.

Dose (Gy)	cells with 1 dicentric	cells with more than 1 dicentric
0.5	0.1758	0.919
1	$1.93e^{-02}$	0.5711
2	$4.64e^{-13}$	0.005668
