# Supplementary material for: Calibration curves by 60Co with low dose rate are different in terms of dose estimation – a comparative study
Source: Genet Mol Biol. 2020 Feb 17;43(1):e20180370. doi: 10.1590/1678-4685-GMB-2018-0370 (PMC7231543; doi:10.1590/1678-4685-GMB-2018-0370)
Supplement: Supplementary file 2 [file 1415-4757-GMB-43-1-e20180370-20200108-suppl2.pdf]

Supplementary Material to “Calibration curves by 60Co with low dose rate are different in terms of dose estimation – a comparative study”

Table S2. Comparison of estimated absorbed doses by selected calibration curves using Dose Estimate software.

**References**	**Bauchinger *et al.* (1983)**	**Schmid *et al.* (2002)**	**This work**	**Martins *et al.* (2013)**	**Lindholm *et al.* (1998)**	**Top *et al.* (2000)**	**Köksal *et al.* (1995)**	**Lloyd *et al.* (1986)**
Country	Germany	Germany	Brazil	Portugal	England	Turkey	Turkey	England
Dose rate (Gy/min) / 0.5 Gy/min	3%	7%	11%	36%	48%	85%	91%	100%
Dicentric frequencies	Estimated doses ± 95% CL (Gy)							
0.02a	0.574 (0.420 0.743)	0.608 (0.438 0.800)	0.566 (0.415 0.732)	0.530 (0.387 0.687)	0.486 (0.354 0.633)	0.511 (0.383 0.653)	0.394 (0.282 0.521)	0.421 (0.310 0.545)
0.15b	1.784 (1.632 1.943)	2.002 (1.825 2.186)	1.739 (1.592 1.891)	1.648 (1.507 1.794)	1.540 (1.407 1.678)	1.519 (1.392 1.651)	1.310 (1.194 1.431)	1.311 (1.199 1.428)
0.2c	2.079 (1.927 2.236)	2.345 (2.167 2.528)	2.022 (1.876 2.174)	1.919 (1.779 2.064)	1.797 (1.664 1.934)	1.764 (1.637 1.895)	1.535 (1.419 1.655)	1.528 (1.416 1.644)
0.7d	3.991 (3.511 4.501)	4.574 (4.013 5.169)	3.863 (3.400 4.353)	3.982 (3.238 4.151)	3.469 (3.048 3.915)	3.353 (2.954 3.777)	2.998 (2.630 3.389)	2.940 (2.585 3.316)
0.75e	4.136 (3.655 4.644)	4.743 (4.181 5.336)	4.002 (3.539 4.491)	3.815 (3.371 4.283)	3.595 (3.174 4.040)	3.473 (3.073 3.896)	3.109 (2.740 3.498)	3.047 (2.691 3.422)
1f	4.793 (4.312 5.298)	5.510 (4.949 6.100)	4.634 (4.172 5.120)	4.421 (3.977 4.886)	4.170 (3.750 4.612)	4.020 (3.620 4.439)	3.613 (3.244 4.000)	3.533 (3.177 3.906)

(95% CL) 95% Confidence limits from combined Poisson and calibration curves errors. "Method A." IAEA 2001; (a) 20 dic/1000cells; (b)150 dic/1000 cells; (c) 200 dic/1000 cells; (d) 70 dic/100 cells; (e) 75 dic/100 cells; (f) 100 dic/100 cells;
