# Supplementary material for: Calibration curves by 60Co with low dose rate are different in terms of dose estimation – a comparative study
Source: Genet Mol Biol. 2020 Feb 17;43(1):e20180370. doi: 10.1590/1678-4685-GMB-2018-0370 (PMC7231543; doi:10.1590/1678-4685-GMB-2018-0370)
Supplement: Supplementary file 3 [file 1415-4757-GMB-43-1-e20180370-20200108-suppl3.pdf]

Supplementary Material to "Calibration curves by 60Co with low dose rate are different in terms of dose estimation – a comparative study"

Table S3. Comparison results of estimated absorbed doses by ANOVA after jackknife-like resampling method.

		Identification number of groups compared											
		1	2	3	4	5	6	7	8	9	10	11	12
Dose rate (Gy/min) /0.5 Gy/min	3%	Bauchinger *et al.* (1983)	Bauchinger *et al.* (1983)						Bauchinger *et al.* (1983)	Bauchinger *et al.* (1983)	Bauchinger *et al.* (1983)	Bauchinger *et al.* (1983)	Bauchinger *et al.* (1983)
	7%	Schmid *et al.* (2002)											
	11%	This work	This work	This work					This work	This work	This work	This work	This work
	36%	Martins *et al.* (2013)	Martins *et al.* (2013)	Martins *et al.* (2013)	Martins *et al.* (2013)				Martins *et al.* (2013)	Martins *et al.* (2013)	Martins *et al.* (2013)	Martins *et al.* (2013)	
	48%	Lindholm *et al.* (1998)	Lindholm *et al.* (1998)	Lindholm *et al.* (1998)	Lindholm *et al.* (1998)	Lindholm *et al.* (1998)			Lindholm *et al.* (1998)	Lindholm *et al.* (1998)	Lindholm *et al.* (1998)		
	85%	Top *et al.* (2000)	Top *et al.* (2000)	Top *et al.* (2000)	Top *et al.* (2000)	Top *et al.* (2000)	Top *et al.* (2000)		Top *et al.* (2000)	Top *et al.* (2000)			
	91%	Köksal *et al.* (1995)	Köksal *et al.* (1995)	Köksal *et al.* (1995)	Köksal *et al.* (1995)	Köksal *et al.* (1995)	Köksal *et al.* (1995)	Köksal *et al.* (1995)	Köksal *et al.* (1995)				
	100%	Lloyd *et al.* (1986)	Lloyd *et al.* (1986)	Lloyd *et al.* (1986)	Lloyd *et al.* (1986)	Lloyd *et al.* (1986)	Lloyd *et al.* (1986)	Lloyd *et al.* (1986)					
		p-values by ANOVA											
Frequencies of dicentrics	0.02	0.646	0.666	0.651	0.677	0.66	0.514	0.793	0.708	0.947	0.898	0.94	0.961
	0.15	0.003*	0.016*	0.029*	0.056	0.134	0.156	0.995	0.0535	0.342	0.442	0.698	0.807
	0.2	<0.001*	0.004*	0.007*	0.031*	0.093	0.121	0.948	0.017*	0.173	0.279	0.561	0.91
	0.7	0.018*	0.089	0.128	0.173	0.366	0.426	0.856	0.197	0.523	0.668	0.894	0.795
	0.75	0.014*	0.083	0.133	0.21	0.343	0.408	0.847	0.187	0.502	0.627	0.772	0.788
	1	0.005*	0.0424	0.0768	0.131	0.256	0.338	0.807	0.118	0.4	0.553	0.728	0.761

(*) p-value < 0.05 means that the estimated doses are not statistically similar.
