# Supplementary material for: Calibration curves by 60Co with low dose rate are different in terms of dose estimation – a comparative study
Source: Genet Mol Biol. 2020 Feb 17;43(1):e20180370. doi: 10.1590/1678-4685-GMB-2018-0370 (PMC7231543; doi:10.1590/1678-4685-GMB-2018-0370)
Supplement: Supplementary file 4 [file 1415-4757-GMB-43-1-e20180370-20200108-suppl4.pdf]

Genetics and Molecular Biology 42, 2, (2020)

Supplementary Material to “Calibration curves by 60Co with low dose rate are different in terms of dose estimation – a comparative study”

Table S4. Results of Tukey´s test after compared all possible pairs of means.

Tukey multiple comparisons of means		Bauchinger *et al.* (1983) versus Köksal *et al.* (1995)	Bauchinger *et al.* (1983) versus Lloyd *et al.* (1986)	Schmid *et al.* (2002) versus Lindholm *et al.* (1998)	Schmid *et al.* (2002) versus Top *et al.* (2000)	Schmid *et al.* (2002) versus Köksal *et al.* (1995)	Schmid *et al.* (2002) versus Lloyd *et al.* (1986)	This work versus Köksal *et al.* (1995)	This work versus Lloyd *et al.* (1986)	Martins *et al.* (2013) versus Köksal *et al.* (1995)	Martins *et al.* (2013) versus Lloyd *et al.* (1986)
Dose rate (Gy/min)/0.5 Gy/min		3% - 91%	3% - 100%	7% - 48%	7% - 85%	7% - 91%	7% - 100%	11% - 91%	11% - 100%	36% - 91%	36% - 100%
Frequencies of dicentrics	Identification number of groups compared	p-values by Tukey’s test									
0.15	1					0.004	0.004				
	2	0.042	0.042								
	3							0.051	0.051		
	4	0.037	0.034	0.036	0.024	0.001	0.001	0.048	0.044		
	8	0.016	0.014					0.021	0.019		
0.20	1							0.012	0.011		
	2									0.056	0.051
	3	0.02						0.025			
	4					0.025	0.019				
	8					0.019	0.014				
0.7	1				0.049	0.008	0.005				
0.75	1		0.07								
1	1					0.004	0.004				
	2	0.042	0.042								

Note. Here were only shown the comparisons of curves in Tukey's test that has a p-value <0.05. Therefore it is the doses that differ statistically and had p-value <0.05 in the ANOVA test shown in table S3.
